# UK trial of pressurised intraperitoneal aerosolised chemotherapy (PIPAC) with oxaliplatin for colorectal cancer peritoneal metastases (NCT03868228)

**DOI:** 10.1515/pp-2023-0008

**Published:** 2023-09-11

**Authors:** Peter Kyle, Kitrick Perry, Anne Moutadjer, Nicholas Gilfillan, Rosalind Webb, Dolan Basak, Paul Ziprin, Dominic Blunt, James Burn, Katherine Van Ree, Antoni Sergot, Jamie Murphy

**Affiliations:** Department of Surgery and Cancer, Imperial College London, London, UK; Imperial College Healthcare NHS Trust, London, UK; Imperial College London, London, UK

**Keywords:** colorectal cancer, peritoneal metastases, pipac, pressurised intraperitoneal aerosolised chemotherapy

## Abstract

**Objectives:**

This is the first UK trial of pressurised intraperitoneal aerosolised chemotherapy (PIPAC) for colorectal cancer peritoneal metastases. This trial aimed to assess the impact of PIPAC in combination with standard of care systemic treatment on: progression free survival (PFS); quality of life (QoL); and short-term complications. In addition, this trial set out to demonstrate that PIPAC can be performed safely in operating theatres within a National Health Service (NHS) setting.

**Methods:**

Single-centre clinical trial with prospective data collection for patients undergoing 8-weekly PIPAC with oxaliplatin at 92 mg/m^2^ from January 2019 till January 2022. Progression free survival was assessed using peritoneal carcinomatosis index (PCI) by CT scans and laparoscopy. Quality of life was assessed by EORTC QLQ-C30 questionnaire. Adverse events were recorded using CTCAE.

**Results:**

Five patients underwent a total of ten PIPAC administrations (median 2, range 1–4). Median PFS was 6.0 months. QoL was maintained across repeat PIPAC procedures but a decrease in social functioning and increased fatigue were evident. Three incidences of grade 3 adverse events occurred but PIPAC was well tolerated.

**Conclusions:**

The presented data demonstrates that PIPAC is feasible and can be safely delivered within the NHS for patients with colorectal cancer peritoneal metastases, but caution must also be exercised given a risk of adverse events. Systemic chemotherapy can be safely administered at a different unit to the PIPAC procedure if both groups have clear lines of communication and timely data sharing.

## Introduction

While significant progress has been made in (neo)-adjuvant therapies for various cancers, extensive colorectal cancer (CRC) peritoneal metastases (PM) continue to be associated with a poor prognosis [1]. The incidence of PM is difficult to quantify particularly due to the limited sensitivity of cross-sectional imaging [2]. The incidence of synchronous PM in colorectal cancer is estimated at 5–10 % but increases to 20–50 % in the metachronous setting [3].

Following a diagnosis of PM, patients with low volume disease amenable to complete excision may be offered cytoreductive surgery (CRS) ± hyperthermic intraperitoneal chemotherapy (HIPEC). For well selected patients CRS is associated with a survival benefit over and above that offered by systemic chemotherapy alone, although the role of HIPEC has been questioned [4]. Despite the potential benefit in survival offered by CRS ± HIPEC it is associated with a considerable morbidity profile and a risk of mortality [5]. As such there is an unmet need for less invasive yet effective treatments for patients with extensive PM, or those with more limited disease who are not candidates for CRS ± HIPEC.

Pressurised intraperitoneal aerosol chemotherapy (PIPAC) is a technique that delivers chemotherapy regimens as a pressurised aerosol into the peritoneal cavity by laparoscopy [6]. Initial reports demonstrated PIPAC achieves significant penetration of chemotherapy agents into PM [7], [8], [9]. Existing studies have found PIPAC to be relatively safe and well tolerated [10], [11], [12] and this conclusion has been reinforced by a recent international consensus statement [13]. PIPAC has been used to treat multiple cancer types, but the evidence base for its use in colorectal cancer PM remains limited [14].

This is the first UK trial of PIPAC and it focused exclusively on colorectal cancer PM. The trial objectives were to assess the impact of PIPAC in combination with standard of care systemic chemotherapy on: 1) progression free survival; 2) quality of life (QoL); and, 3) short-term complications. In addition, this trial set out to demonstrate that PIPAC can be performed safely in operating theatres within a National Health Service (NHS) setting.

## Subjects and methods

### Trial design

**Figure 1:**
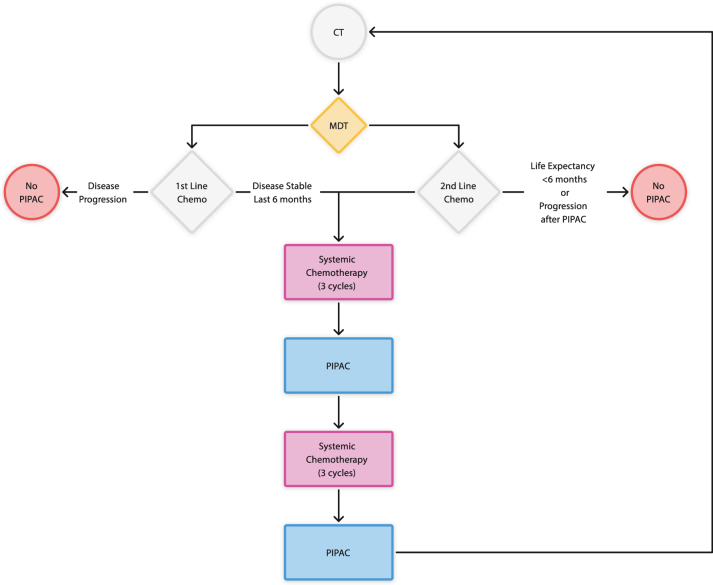
Trial protocol flowchart.

This trial was a prospective single centre clinical trial assessing PIPAC in combination with standard of care systemic chemotherapy for colorectal cancer PM. Patients received standard of care systemic chemotherapy as deemed appropriate by their local oncology service which was synchronised with delivery of PIPAC on an 8-weekly basis, replacing a cycle of systemic therapy at that time point (Figure 1). Ethical approval was granted by a Research Ethics Committee of the Health Research Authority (Reference: 18/LO/1610) and the trial was registered with an online clinical trials registry (ClinicalTrials.gov; NCT03868228). The data presented below describes consecutive patients treated between January 2019 and January 2022, including an 18-month pause in the trial due to the COVID-19 pandemic.

The primary outcome measure was progression free survival as assessed by cross-sectional imaging prior to PIPAC administration, or at the time of laparoscopy, with the point of progression defined as the first of these investigations demonstrating an increase in peritoneal carcinomatosis index (PCI) [15]. Secondary outcome measures were: 1) QoL as assessed by EORTC QLQ-C30 [16]; 2) serious adverse events or operative complications as defined by CTCAE (Version 5); and 3) PIPAC related safety regulation breaches/adverse events during administration in operating theatre.

### Patient selection

Patients with colorectal cancer PM were identified from cases discussed at the peritoneal multi-disciplinary team (MDT) meetings between January 2019 and January 2022. Those who were deemed not to be candidates for CRS ± HIPEC by the MDT due to disease burden or patients who declined that treatment option, were considered for eligibility into the trial.

Inclusion criteria were defined as: 1) patients with CRC PM with life expectancy of >6 months; 2) ECOG performance status (PS) 0 or 1; 3) systemic chemotherapy regimens compatible with 8-weekly PIPAC administration or without systemic chemotherapy if no systemic options were available; 4) MDT agreement that the patient was a suitable candidate for PIPAC. Initially, a 15-mile catchment area inclusion criterion was applied due to concerns regarding the safety of a patient receiving systemic chemotherapy and PIPAC delivery by different oncology services. However, following a positive experience with the first two trial patients this was reviewed and adapted. Consequently, a trial protocol amendment was approved to expand geographical recruitment providing a satisfactory relationship could be established with the referring oncology service to allow safe PIPAC delivery at Imperial College and ongoing systemic chemotherapy under the care of the patient’s local unit.

Exclusion criteria were defined as: 1) systemic chemotherapy regimens not compatible with 8-weekly PIPAC administration (trifluridine/tipiracil, or bevacizumab); 2) clinically evident gross ascites; 3) bowel obstruction; 4) previous bone marrow suppression due to chemotherapy that did not sufficiently respond to granulocyte colony-stimulating factor (due to the risk of post-operative neutropenia). Low volume distant metastases, such as in the lung or liver, were not considered an absolute contraindication. On a case-by-case basis, the MDT assessed whether the peritoneal metastases were felt to be the life limiting site of disease and thus targeted peritoneal treatment may increase overall survival.

### Coordination of systemic chemotherapy and PIPAC

The scheduling and delivery of standard of care systemic chemotherapy regimens with 8-weekly PIPAC delivery required close collaboration between the oncology, pharmacy, and surgical teams. The process was coordinated and facilitated by a clinical nurse specialist (AM) and clinical research fellow (PK). In cases where systemic chemotherapy was not delivered at Imperial College, extra steps were required to ensure timely data sharing between units and a defined pathway was used. Granulocyte colony stimulating factor was routinely administered for 3 days following systemic chemotherapy administration to reduce the risk of neutropenia, as this would delay PIPAC or substantially increase the risk of complications surgery.

The exact timing of PIPAC was dependent upon the systemic chemotherapy regimen duration, the day of the week systemic chemotherapy was delivered and operating theatre availability. For patients receiving 2-weekly systemic chemotherapy regimens there was an approximately 14-day gap between systemic chemotherapy and PIPAC, equating to PIPAC administration in place of every fourth systemic chemotherapy cycle on an 8-weekly PIPAC regimen. Prior to PIPAC delivery preoperative blood tests were taken and an oncology telephone appointment was scheduled to review the preceding systemic chemotherapy dates, blood test results, lack of exclusion criteria, and to prescribe intraperitoneal oxaliplatin at 92 mg/m^2^ (KP & DB). A baseline computed tomography (CT) scan of the thorax, abdomen and pelvis with intravenous contrast was required within 6 weeks prior to first PIPAC administration and following every two PIPAC procedures thereafter. CT scans were assessed for radiological PCI scores by a Consultant Radiologist with a specialist interest in gastrointestinal radiology. All CT scans were reviewed as part of the MDT meeting.

### Data collection

The following data points were collected as part of the trial: American Society of Anesthesiology (ASA) score; Eastern Cooperative Oncology Group (ECOG) performance status (PS); incidence of failed entry at laparoscopy; PCI score; intraoperative complications or safety adverse events relating to PIPAC delivery in the operating theatre; length of hospital stay; 90-day post-operative adverse events according to the Common Terminology Criteria for Adverse Events classification version 5.0 (CTCAE). Adverse events were recorded contemporaneously within the medical records by the treating clinicians and retrospectively graded. Quality of life was assessed at baseline and just before each PIPAC procedure using the validated European Organization for Research and Treatment of Cancer (EORTC) generic questionnaire QLQ-C30 (version 3.0). The analyses provided results in three domains: 1) global health status; 2) functional scales; 3) symptom/item scales. In the global health status and functional scales, a higher score indicates a higher QoL and functional status but conversely a lower score in the symptom scale is considered positive as it reflects a lower level of symptomatology.

### PIPAC procedure

The trial surgeons (JM & PZ) and anaesthetists (NG) completed the International Society of Surgery for Pleura and Peritoneum training course prior to this trial commencing. PIPAC was delivered in line with this training and the standardised technique has been previously described in the literature [17]. Our group performed an extra step to achieve an airtight seal at the site of open cut down for the first 12 mm laparoscopic balloon port (Applied Medical, Düsseldorf, Germany) by using a 2-0 nylon suture to create a temporary skin purse string, in addition to the standard fascial stay sutures. The other balloon port(s) that were inserted under direct laparoscopic vision did not require a skin purse string suture. Pneumoperitoneum of 12 mmHg was established, the PCI score was assessed, and peritoneal biopsies taken. A safety checklist was completed and the operating theatre cleared of personnel. Oxaliplatin was delivered through the CapnoPen^®^ (Capnomed GmbH, Villigendorf, Germany) by remotely controlled high pressure injector (ProVis Mark V, Medrad, Bayer, Germany), allowing an additional 30 min after delivery before re-entry to the operating theatre, and then controlled gas evacuation into a closed aerosol waste system and closure of the wounds. The patient was managed post-operatively with contact precautions in place and by using the personal protective equipment (PPE) necessary for potential chemotherapy skin or surface contamination.

## Results

Five patients were recruited and the demographics and baseline characteristics are outlined in Table 1. All patients had the cancer primary *in situ*. Two patients had synchronous distant metastases. Most patients were heavily pre-treated with systemic chemotherapy with a median of 2 prior regimens (range 1–2). The first four patients had systemic chemotherapy and PIPAC at Imperial College but the final patient had systemic chemotherapy at their local oncology centre and received only PIPAC at Imperial College.

**Table 1:** Demographics and baseline characteristics.

Patient	P1	P2	P3	P4	P5	Median
Age, years	69	38	44	34	57	44
Sex	F	M	M	M	F	
ECOG PS	0	1	1	0	0	0
ASA score	2	3	2	2	3	2
Co-morbidities	Osteoporosis, hypercholesterolaemia	NAFLD	HIV	Pulmonary embolism	Asthma, DVT, Hashimoto’s thyroiditis, hypertension, choledocholithiasis	
Cancer primary	Indeterminate – caecum/appendix	Caecum	Synchronous rectosigmoid and appendix	Sigmoid	Caecum	
Tumour type	Poorly differentiated mucinous adenocarcinoma	Poorly differentiated mucinous adenocarcinoma with signet ring cells	Poorly differentiated mucinous adenocarcinoma with signet ring cells	Poorly differentiated adenocarcinoma	Moderately differentiated mucinous adenocarcinoma	
Other distant metastases	None	Liver, bone (spine)	None	None	Liver	
Previous systemic chemotherapy (no. of lines)	2	2	2	1	2	2

PS, performance status; NAFLD, non-alcoholic fatty liver disease; HIV, human immunodeficiency virus; DVT, deep vein thrombosis.

### Survival and follow up

The first four patients have since died as a result of their cancer. Patient 5 is alive, but the peritoneal metastases progressed during a postponement of a planned third PIPAC. Summary survival data is outlined in Table 2.

**Table 2:** Time from diagnosis to PIPAC and survival.

Patient	Time – diagnosis till PIPAC, months	Progression free survival from first PIPAC, months	Overall survival from first PIPAC, months	Overall survival from diagnosis, months
P1	7.6	25.1	31.6	39.2
P2	2.2	4.5	5.1	7.2
P3	8.7	^a^	4.7	13.4
P4	12.1	0.5	12.9	25.0
P5	30.5	7.6	(11.6)	(42.2)
Median	8.7	6.0	11.6	25.0

( ), patient still alive; ^a^no data available.

Patient 1 died 31.6 months after first PIPAC due to disease progression. Patient 2 died 112 days following a second PIPAC. The death was due to progression of known liver metastases resulting in biliary obstruction. Patient 3 deteriorated physically with a drop in performance status whilst awaiting a second PIPAC and no longer felt able to travel from out of region to have systemic chemotherapy and PIPAC. He died 4.7 months after PIPAC. There is no accurate progression free survival data as he returned to his local hospital. Pre-PIPAC scans did not show gross ascites, but at laparoscopy 5 L was aspirated, this likely indicated interval disease progression. Following an initial PIPAC, patient 4 developed bowel obstruction from the cancer primary, which is a contraindication for PIPAC [18]. No further PIPAC was offered and he died after 12.9 months. Patient 5 remains under follow up and has reached 11.6 months at the time of writing.

### Quality of life (QoL)

**Figure 2:**
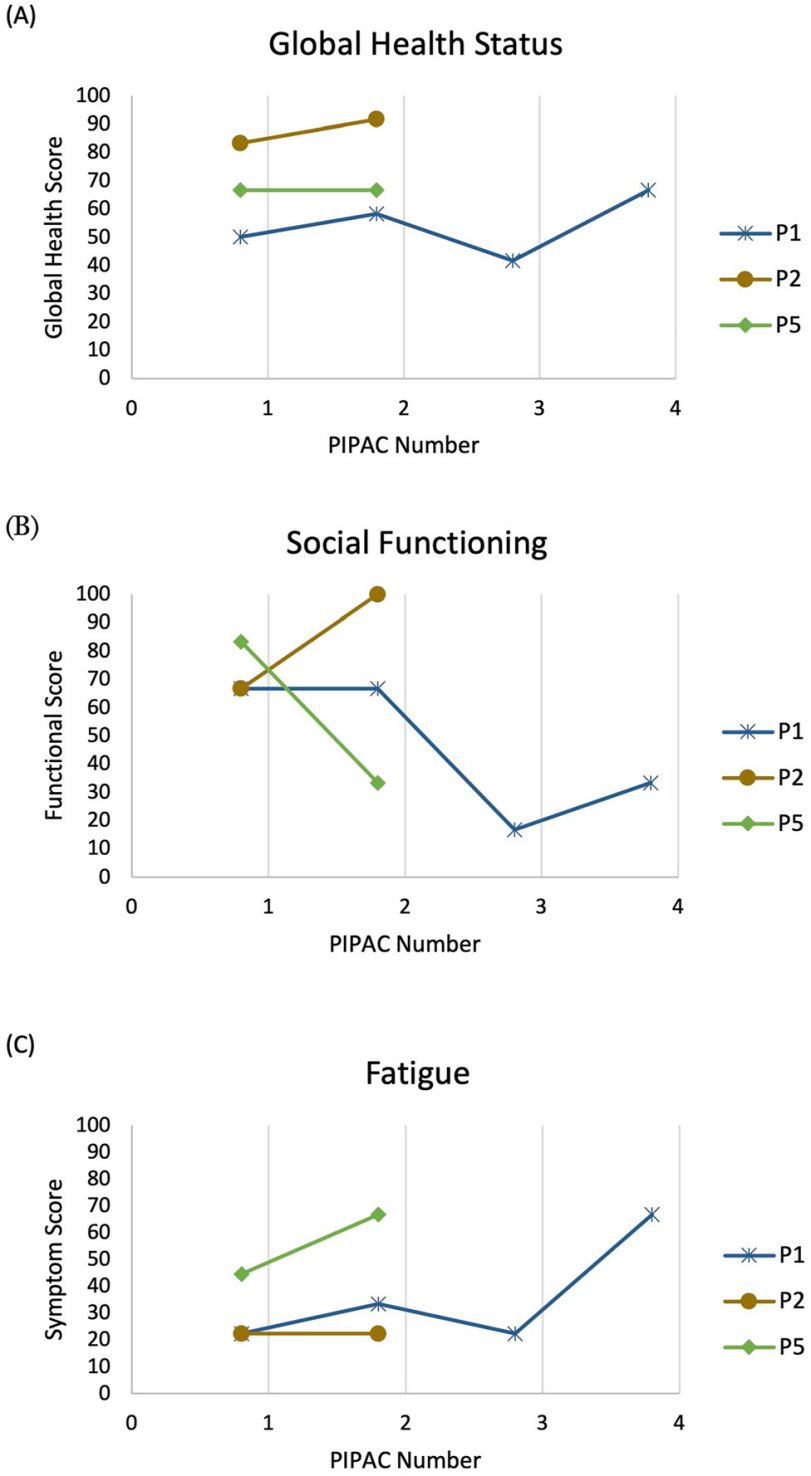
Selected domains from EORTC QLQ-C30 results.

Interpretation of the QoL results is limited due to the small dataset and lack of repeat time points. No statistical assessment can be performed as a result. However, in the studied patients the global health status (Figure 2A) did not show any deterioration following PIPAC. Similarly, across the physical, role, emotional and cognitive functioning scales the scores appeared to be mostly static across time. Within the symptom/item scale results, most domains (nausea & vomiting, pain, dyspnoea, insomnia, appetite loss, constipation, diarrhoea and financial difficulties) showed no clear trend in any direction. The exception was that of patient 1 and patient 5’s social functioning scores which demonstrated a downward trend and inversely an increasing fatigue symptom score (Figure 2B and C).

### Safety and adverse events

There were no intraoperative complications during the trial (Table 3). Across all five patients and ten PIPAC administrations, there were three occurrences of severe adverse events (grade 3) within 90 days of PIPAC delivery. There were no grade 4 or 5 adverse events. All other adverse events were mild to moderate (Grade 1–2: no invasive intervention required) and in keeping with expected side effects of general anaesthesia and laparoscopy (Table 4). There were no instances of biochemical toxicity (hepatic or renal).

**Table 3:** PIPAC procedural data.

Patient	Radiological baseline PCI	PIPAC	Laparoscopic PCI (score/maximum accessible)	Ascites, mL	Failed access, (Y/N)	Intraoperative complication, (Y/N)
P1	>20/39	1	20/27	0	N	N
		2	20/27	20	N	N
		3	21/24	0	N	N
		4	6/6	0	N	N

P2	28/39	1	29/33	800	N	N
		2	26/30	600	N	N
P3	27/39	1	28/39	5,000	N	N
P4	23/39	1	24/36	0	N	N
P5	32/39	1	26/27	0	N	N
		2	19/24	0	N	N

**Table 4:** Procedure and safety data.

Patient	PIPAC	LOS, days	CTCAE adverse events (90-days)				Breakdown
			Grade 1	Grade 2	Grade 3	Grade 4/5	
P1	1	1	3	3	0	0	G1 – distension; constipation; fatigue
							G2 – pain; vomiting; nausea
	2	1	2	2	0	0	G1 – distension; constipation
							G2 – nausea; pain
	3	1	2	1	0	0	G1 – fatigue; constipation
							G2 – pain
	4	1	0	3	0	0	G2 – pain; nausea; constipation
P2	1	1	1	1	0	0	G1 – pain
							G2 – vomiting
	2	0	1	1	0	0	G1 – pain
							G2 – nausea
P3	1	1	3	2	0	0	G1 – fever; distension; nausea
							G2 – pain; thrush
P4	1	2	1	1	1	0	G1 – pain
							G2 – hypotension
							G3 – Febrile neutropenia
P5	1	1	4	1	0	0	G1 – fatigue; pain; mucositis oral; herpes simplex reactivation
							G2 – bronchial infection
	2	4	1	2	2	0	G1 – pain
							G2 – hypotension; nausea
							G3 – hypoxia; bronchial infection
Median	2	1	1.5	1.5	0	0	

Length of stay (LOS) and adverse events as per Common Terminology Criteria for Adverse Events (CTCAE) version 5.0. G1, Grade 1; G2, Grade 2; G3, Grade 3.

After each PIPAC all patients reported Grade 1 or 2 abdominal pain post-operatively. Most (4 of 5 patients across 6 of 10 PIPAC) reported nausea with two patients also experiencing vomiting.

Patient 1 reported post-operative constipation, but this symptom was a chronic symptom which actually became less severe following PIPAC.

Patient 3 had a grade 1 post-operative fever within the first 24 h but this settled without intervention. Patient 4 had low level fevers (maximum 37.8 °C) which did not meet CTCAE criteria (>38 °C) for an adverse event and no antibiotics were required.

Patient 4 was readmitted at their local hospital 16 days following first PIPAC administration with grade 3 febrile neutropenia. On that admission this patient was demonstrated to have obstruction of the defunctioned colon between the sigmoid tumour and a competent ileocaecal valve. This was managed at the patient’s local hospital with intravenous antibiotics and a colonic stent.

Patient 5 suffered a lower respiratory tract infection, on a background of asthma, following the first PIPAC administration. This necessitated a one-week postponement of subsequent systemic chemotherapy administration which in turn pushed back a second PIPAC to 9 weeks. A grade 3 hypoxia and bronchial infection followed the second PIPAC administration. This required an inpatient stay of 4 days with supplemental oxygen and intravenous antibiotics. Respiratory symptoms recurred in the weeks following the second PIPAC and plans for a third were put on hold whilst specialist respiratory input was sought and treatment for possible atypical pneumonia with azithromycin was implemented. Patient 5 improved clinically but during this delay repeat CT demonstrated subtle progression of peritoneal metastases and no further PIPAC was scheduled.

### Occupational health and safety

There was a single episode of spillage of chemotherapy during the trial. A few millilitres were accidentally lost whilst transferring the oxaliplatin from the pharmacy prepared syringes to the high-pressure injector syringe. This was captured by the pre-placed chemotherapy bin below the injector and surrounding disposable floor coverings.

### Assessment of peritoneal disease

**Figure 3:**
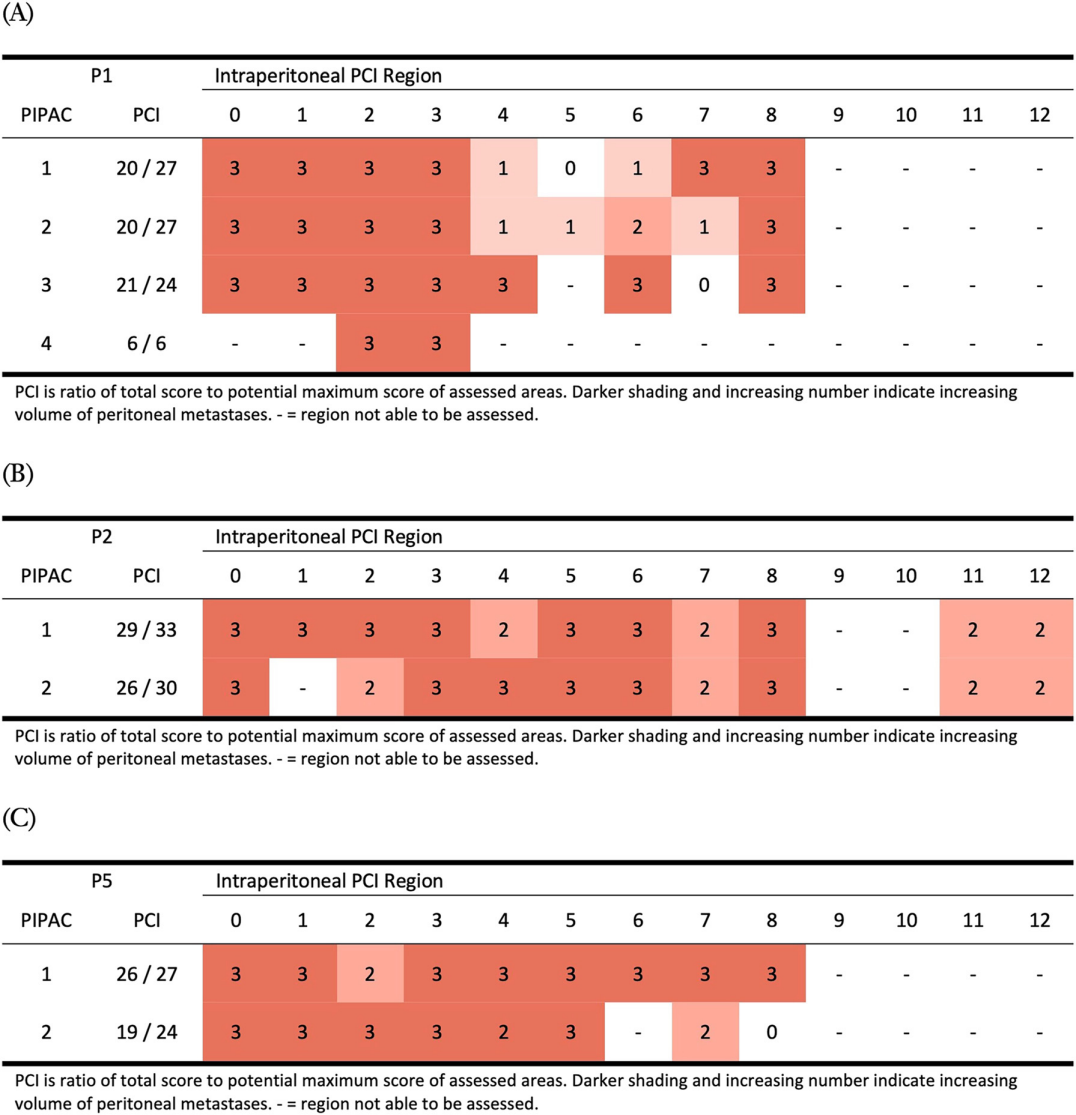
Heatmap of location and volume score of peritoneal metastases across multiple PIPAC administrations.

All PIPAC procedures were performed by a single surgeon (JM) and thus interobserver variability was eliminated. There was no failure of entry at any laparoscopy. For patients who underwent more than one PIPAC, Figure 3 demonstrates laparoscopic PCI score assessment in the form of a heatmap outlining location and volume of PM throughout treatment. The region-specific scores generally remained static with limited evidence of progression or regression of PM. At baseline, visual access to several intra-abdominal regions was limited, particularly the small bowel (regions 9 to 12), as much was obscured by heavily diseased overlying omentum. At repeat PIPAC further regions were inaccessible due to progressive intra-abdominal adhesions, most clearly demonstrated with patient 1 (Figure 3A). For patient 1, further PIPAC administration beyond a fourth was deemed not feasible due to these adhesions.

Peritoneal disease extent was also assessed and monitored by repeat CT scan after two PIPAC procedures. Compared to baseline imaging patient 1 demonstrated stable disease after two PIPAC, after four PIPAC and on five further scans over the following 20 months of follow up but with increasing ascites on the final scan. Patient 2 showed stable peritoneal disease after two PIPAC but progression of known liver metastases thus no further PIPAC was offered as per trial protocol.

Patient 5 also demonstrated stable disease but an additional later CT, conducted whilst awaiting a postponed third PIPAC, showed subtle peritoneal progression. Patients 3 and 4 only had one PIPAC thus no CT reassessment was made.

### Length of stay (LOS)

The median LOS following PIPAC administration was 1 day (range 0–4) (Table 3). Initially the overnight stays were due to caution given PIPAC was a new technique to the surgeons, hospital, and United Kingdom. As the team became more comfortable with PIPAC and the safety profile of the technique, the trial team were satisfied that a day case approach was possible.

## Discussion

The small number of recruited patients (n=5) in this trial does not permit an adequate assessment of impact upon survival. In keeping with existing studies, QoL appears maintained across repeat PIPAC administration [19] but this trial showed there may be an increase in fatigue and a drop in social functioning, although it is unclear if this is due to PIPAC administration alone, or in combination with ongoing systemic chemotherapy.

Taking frequency of adverse events, this trial reports a rate of 30 % for grade 3 (3 instances across 10 PIPAC) and zero grade 4 and 5. A Recent systematic review of PIPAC in CRC PM found a similar rate of adverse events with 19.0 % Grade 3 and 3 % grade 4 [20]. A broader review covering multiple cancer primary types, describes a lower pooled grade 3 adverse event incidence of 7 %, grade 4 0.8 % and grade 5 1.6 % [21]. However, in both reviews, many studies did not include sufficient breakdown of data to allow for pooled analysis. Reporting also varied across studies as often only the most severe adverse event was reported, as opposed to frequency of all adverse events as reported here.

This trial demonstrates that PIPAC is feasible and can be safely delivered within the NHS for patients with colorectal cancer peritoneal metastases, but caution must be exercised given a risk of adverse events in this patient group. Scheduling intercalated PIPAC within a systemic chemotherapy regimen requires significant coordination from multiple teams and benefits from dedicated staff support. The scheduling must also be sufficiently adaptable to allow for unplanned delays to chemotherapy. Systemic chemotherapy can be safely administered at a different unit to the one delivering PIPAC if both groups have good lines of communication and timely data sharing.

This trial demonstrated that laparoscopic assessment of PCI in patients with extensive disease is challenging due to visually inaccessible areas. Additionally, repeated PIPAC with oxaliplatin appeared to promote significant adhesion formation and fibrosis, further limiting visual PCI assessment and potentially impaired distribution of the aerosolised therapeutic agent. At repeat laparoscopy, visual assessment also struggled to delineate between definite metastases and possible fibrosis which has been postulated to be a mechanism of action associated with Oxaliplatin via PIPAC [22].

The major limitation of this trial was the low recruitment number, and the COVID-19 pandemic was a major factor as this resulted in the trial being paused for 18 months. Furthermore, narrow inclusion criteria also limited recruitment. The novel nature of PIPAC and the current limited evidence base for efficacy resulted in the trial group being extremely cautious with patient selection. In particular, there was concern regarding interruption of systemic chemotherapy regimens to enrol patients for experimental and invasive treatments, which might also cause oncological harm either from a complication that delayed systemic treatment, or simply from the breaks in systemic therapy that PIPAC administration requires. For that reason the trial team included a criterion requiring that sequential cross-sectional imaging must show stable disease for a period of 6 months prior to a patient being considered for PIPAC, which in itself is a potential source of bias in the reported results.

Future studies, particularly those with standard of care comparator arms, will be better placed to assess the efficacy of PIPAC, expand inclusion criteria and potentially offer PIPAC earlier in the treatment pathway of patients with peritoneal metastases before performance status declines. Future studies would also benefit from systemic chemotherapy being delivered by units local to the patient as this would significantly reduce the travel burden and cost for patients, in addition to potentially increasing access to PIPAC treatment. More PIPAC delivery sites across the UK would also improve recruitment to future trials. Primary outcome measures for future studies may need to look at peritoneal specific progression free survival given PIPAC is a targeted intervention to the peritoneum, with extraperitoneal sites of disease being treated by other integrated modalities. To facilitate this approach, novel standardised radiological methods of assessing peritoneal disease burden and response may need to be developed. The effect of PIPAC upon patients QoL was not clearly determined by this trial. In order to gain greater insight into this matter, larger cohorts with assessments at more frequent time points will be needed. The National Institute for Health and Care Excellence review (Ref: IPG681 [23]) has determined that future use of PIPAC in the UK should be undertaken within randomized controlled trials and many of the points above will be addressed by a national, multi-centre, randomised trial (PICCOS) set to begin in 2023.
